# Chromium-Induced Ultrastructural Changes and Oxidative Stress in Roots of *Arabidopsis thaliana*

**DOI:** 10.3390/ijms160715852

**Published:** 2015-07-13

**Authors:** Eleftherios P. Eleftheriou, Ioannis-Dimosthenis S. Adamakis, Emmanuel Panteris, Maria Fatsiou

**Affiliations:** Department of Botany, School of Biology, Aristotle University of Thessaloniki, 54124 Thessaloniki, Greece; E-Mails: iadamaki@bio.auth.gr (I.-D.S.A.); epanter@bio.auth.gr (E.P.); mfatsiou@windowslive.com (M.F.)

**Keywords:** *Arabidopsis thaliana*, Cr(VI) toxicity, electron dense deposits, oxidative stress, ultrastructural effects

## Abstract

Chromium (Cr) is an abundant heavy metal in nature, toxic to living organisms. As it is widely used in industry and leather tanning, it may accumulate locally at high concentrations, raising concerns for human health hazards. Though Cr effects have extensively been investigated in animals and mammals, in plants they are poorly understood. The present study was then undertaken to determine the ultrastructural malformations induced by hexavalent chromium [Cr(VI)], the most toxic form provided as 100 μM potassium dichromate (K_2_Cr_2_O_7_), in the root tip cells of the model plant *Arabidopsis thaliana*. A concentration-dependent decrease of root growth and a time-dependent increase of dead cells, callose deposition, hydrogen peroxide (H_2_O_2_) production and peroxidase activity were found in Cr(VI)-treated seedlings, mostly at the transition root zone. In the same zone, nuclei remained ultrastructurally unaffected, but in the meristematic zone some nuclei displayed bulbous outgrowths or contained tubular structures. Endoplasmic reticulum (ER) was less affected under Cr(VI) stress, but Golgi bodies appeared severely disintegrated. Moreover, mitochondria and plastids became spherical and displayed translucent stroma with diminished internal membranes, but noteworthy is that their double-membrane envelopes remained structurally intact. Starch grains and electron dense deposits occurred in the plastids. Amorphous material was also deposited in the cell walls, the middle lamella and the vacuoles. Some vacuoles were collapsed, but the tonoplast appeared integral. The plasma membrane was structurally unaffected and the cytoplasm contained opaque lipid droplets and dense electron deposits. All electron dense deposits presumably consisted of Cr that is sequestered from sensitive sites, thus contributing to metal tolerance. It is concluded that the ultrastructural changes are reactive oxygen species (ROS)-correlated and the malformations observed are organelle specific.

## 1. Introduction

Many heavy metals are essential for plant growth and development, but excessive levels of either essential or non-essential metals, such as chromium (Cr), are toxic to plants, causing a wide range of deleterious effects. Being one of the most abundant elements on the earth’s crust, naturally occurring in widespread mafic-ultramafic complexes [1] and possessing some unique physical properties (lustrous, colorful compounds, high corrosion resistance, hardness), Cr is widely used in industry including in metallurgy, electroplating, tannery, manufacturing of paints, pigments and textile dyes, wood preservation and paper production [2]. Due to these human activities, it may accumulate locally at high concentrations, raising concerns for human health hazards [3]. The impact of Cr to environmental systems and organisms depends on its valence or the oxidation state. Most of the Cr in soil occurs in its trivalent state [Cr(III)], which is less mobile, very stable and less toxic as compared to the hexavalent one [Cr(VI)], that is readily soluble, highly mobile, very unstable and more toxic [1]. In traces, the trivalent state is essential for human nutrition [4], but both forms are toxic to living organisms at high concentrations, even carcinogenic [5,6]. However, neither form of Cr is essential for plants and there is no specific mechanism for its uptake [7,8].

The great majority of the studies carried out on the Cr–plants interaction refers to Cr uptake, translocation, accumulation, tolerance, phytoremediation, its effects on seed germination, seedling growth, plant morphology, biomass production, physiological processes, yield and gene induction [7,8]. Chromium has also been shown to exert cytotoxic, genotoxic and mutagenic effects to plant cells, manifested as cell cycle arrest, impairment of cell division dynamics, prominent chromosomal abnormalities, induction of micronuclei formation and repression of antioxidative enzymes [9,10,11]. Aberrations of the mitotic division were correlated with and attributed to the Cr-induced derangement of the microtubule cytoskeleton, which normally underlies cell division [12,13,14]. The extent of DNA damage depends on the time of exposure and the concentration of Cr(VI), while enhancement of micronuclei formation is considered an indication of clastogenicity [9].

On the other hand, studies on the effects of Cr(VI) on plant morphology and cell fine structure are limited and interspersed among unrelated species, revealing different or even contradictory findings. Mitochondria and plastids are among the most frequently reported organelles to be deranged under Cr stress. In different plants treated with Cr(VI) at concentrations ranging from 50 to 1000 μM for exposure periods extending from a few hours up to 90 days, mitochondria displayed ruptured outer membranes, altered internal cristae, swollen and spherical morphology or accumulation of electron dense material [15,16,17,18]. More recently, it was shown that mitochondria of 400 μM Cr-treated seedlings of *Brassica napus* remained undeveloped even after 15 days of exposure compared to control [19].

Plastids, in particular chloroplasts, became contracted and spherical in the presence of Cr(VI) [15,18,19,20]. A concentration and time-dependent distortion was also recognized in *Spirodela polyrhiza*, where chloroplasts became spherical with very irregular contour, contained large starch grains and/or plastoglobuli, and their volume was reduced to less than half of the control samples [16]. Other frequently reported effects of Cr stress refer to membrane injury, vacuole disruption and cytoplasm dilution due to mixing with vacuolar contents [15,16,18,21].

As most of the above studies were environmentally- and/or agriculturally-focused, studies on the cellular effects of Cr(VI) on a model plant are quite scarce. In particular, data about the effects of Cr(VI) to the fine structure of *Arabidopsis thaliana* are missing. Root hairs and biomass production in *A. thaliana* were stimulated by 100 μM Cr(VI), but concentrations greater than 200 μM were toxic causing arrest of plant growth, leaf chlorosis and disruption of root system architecture [22]. Morphological root changes may be part of a phytohormonal response, common against both biotic and abiotic stresses [23]. By using Petri dishes with a gradient of distance between germinating seeds and a cadmium/copper/zinc-contaminated medium at sub-toxic concentrations, a remodeling of *A. thaliana* root architecture was observed and was interpreted as a pollution “escaping strategy” aiming at seeking metal-free patches [24].

Considering the extensive range of plant responses to Cr and the scarcity of data concerning its toxicity to a model plant, the present study was undertaken aiming to thoroughly elucidate the ultrastructural alterations of *A. thaliana* root tip cells exposed to potassium dichromate (K_2_Cr_2_O_7_, Cr(VI) for brevity), in comparison to those of other species. A key question was to elucidate whether all cell organelles and compartments were affected to the same extent. Moreover, the effects of Cr(VI) to cell vitality, callose deposition, reactive oxygen species (ROS) production and peroxidase activity were also investigated in order to correlate them with any ultrastructural malformations. The model plant *A. thaliana* [25] is fit for such a study, as the root architecture and typical ultrastructure are well known, its delicate roots are ideal for whole mount observations and it grows readily in artificial conditions. Our results indicate that the Cr(VI)-induced ultrastructural changes seem to be organelle-specific and correlated to oxidative stress.

## 2. Results

### 2.1. Morphological Effects, Reactive Oxygen Species (ROS) Disturbance and Cell Mortality

**Figure 1:**
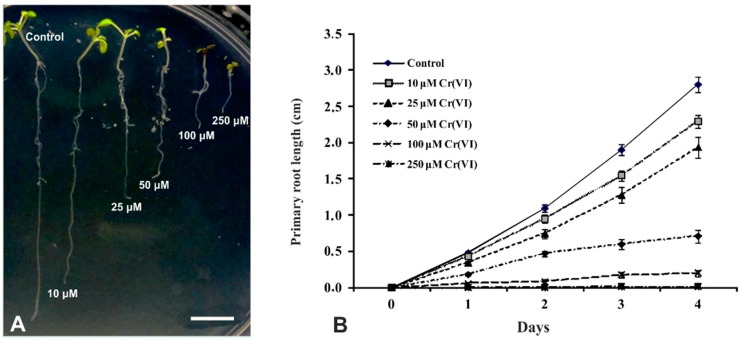
(**A**) Representative seedlings of *Arabidopsis thaliana*, first germinated in water for 4 days and then exposed for another 4 days to water (control) and increasing concentrations of Cr(VI). Note the decreasing size of the plantlets upon increasing Cr(VI) concentration; (**B**) Difference of primary root lengthening of 4-day-old pre-germinated seedlings of *A. thaliana* (day zero) exposed to water (control) and 10, 25, 50, 100 and 250 μM Cr(VI) solutions. Each point represents the average ± standard error of a sample size *n* = 30. Scale bar: 1 cm. When different optical sections or different magnifications of the same specimen are used, they are identified with the same letter and numbered consecutively. In all figures except for Figure 1, seedlings were exposed to 100 μM potassium dichromate (K_2_Cr_2_O_7_, Cr(VI) for brevity) for periods as depicted on the individual panels.

Four-day-old seedlings of *A. thaliana* exposed to graded concentrations of Cr(VI) for four additional days displayed a concentration-dependent decrease of primary root and shoot length compared to the control (Figure 1A). The quantification of primary root growth rate, determined by measuring of root length of 30 seedlings per treatment every 24 h for four consecutive days (Figure 1B), reflected the macroscopic evidence.

**Figure 2:**
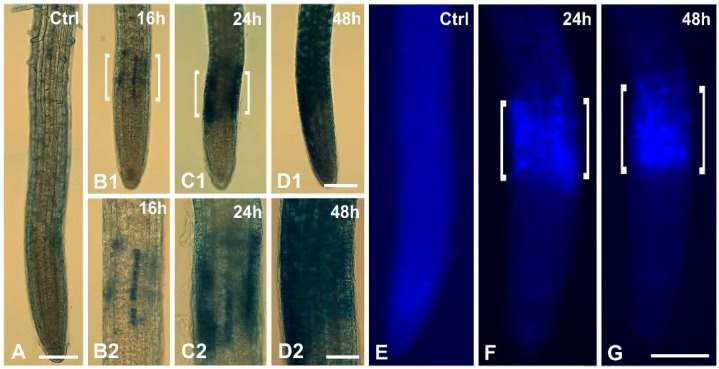
Detection of cell death (by Evans blue staining) and callose deposition in root tips of *A. thaliana*. (**A**–**D**) Evans blue staining of an untreated (**A**) and Cr(VI)-treated roots (**B**–**D**), at lower (**B1**–**D1**) and higher (**B2**–**D2**) magnifications. Note the time-dependent enhancement of staining, especially at the transition zone (**B**–**D**, brackets); (**E**–**G**) Callose detection with aniline blue; (**E**) Control. No specific fluorescence is seen; (**F**,**G**) 24 h (**F**) and 48 h (**G**) Cr(VI) treatment. Aniline blue fluorescence is localized in the transition zone (brackets). Scale bars: (**A**,**B1**–**D1**,**E**–**G**): 100 μm; (**B2**–**D2**): 50 μm.

Cell mortality of 100 μM Cr(VI)-treated roots was determined with Evans blue and Trypan blue staining. In untreated seedlings no Evans blue staining was observed (Figure 2A). After 16 h of Cr(VI) exposure, staining was detected in some centrally located cells, especially at the transition zone (Figure 2B1,B2), which was more extensive after 24 h of treatment (Figure 2C1,C2). After 48 h of Cr(VI) exposure the staining was widespread (Figure 2D1,D2). The Trypan blue experiment produced the same results as Evans blue staining (data not shown).

Callose deposition was analyzed with aniline blue staining. In untreated roots no specific aniline blue signal was observed, except for a vague background (Figure 2E), suggesting the lack of callose deposition. On the other hand, in Cr(VI)-treated roots intense aniline blue fluorescence was localized in the transition zone (Figure 2F,G), roughly coinciding spatially with Evans blue staining (*cf.* Figure 2B–D).

**Figure 3:**
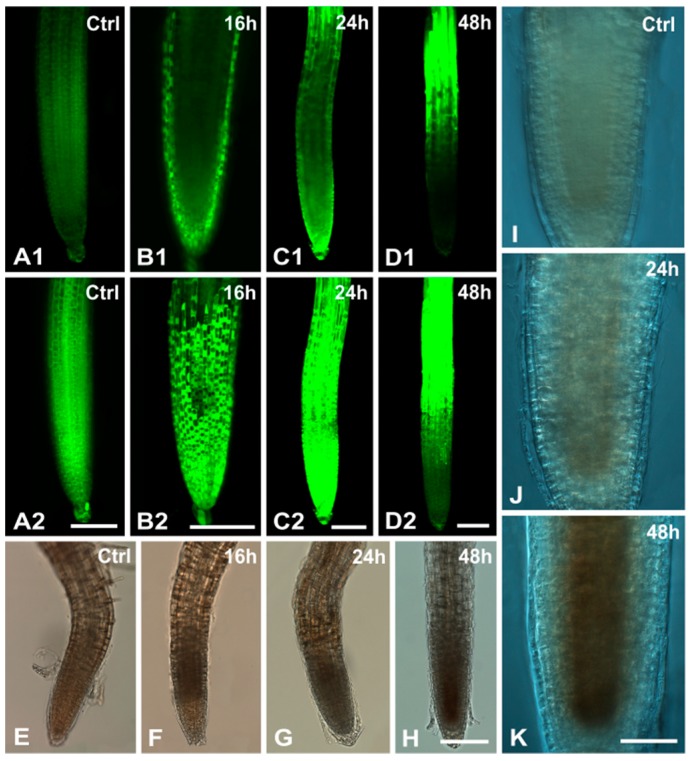
Visualization of H_2_O_2_ concentration and peroxidase activity in root tips of *A. thaliana*. (**A**–**D**) 2,7-dichlorofluorescein diacetate (DCF-DA) staining of control (**A**) and variously Cr(VI)-treated roots (**B**–**D**) at single median CLSM sections (designated as 1) and maximum projections of serial CLSM sections (designated as 2). Note the intensification of the fluorescent signal after a 16 and 24 h effect, primarily localized in the undifferentiated root zone, indicative of increasing H_2_O_2_ accumulation; (**E**–**K**) Detection of peroxidase activity in Cr(VI)-treated roots by pyrogallol staining in bright field at low magnifications of whole mounts (**E**–**H**) and higher magnifications of centrally focused images under differential interference contrast optics (**I**–**K**); An increasing peroxidase activity occurs in a time-of-exposure dependent manner (**E**–**H**), localized mainly in the internal root tissues (**I**–**K**). Scale bars: (**A**–**H**): 100 μm; (**I**–**K**): 50 μm.

The production of H_2_O_2_, detected with the DCF-DA marker, showed a low signal in control roots (Figure 3A). In Cr(VI)-treated seedlings, a time-dependent intensification of fluorescence was observed, especially at the 16 and 24 h applications, indicating an increasing concentration of H_2_O_2_ (Figure 3B,C). Comparison of single central optical sections (Figure 3B1–D1) with maximum projections of the respective roots (Figure 3B2–D2) revealed that in 16 and 24 h applications most of H_2_O_2_ was localized in the undifferentiated root zone, while after 48 h most of the H_2_O_2_ was localized in the differentiated root zone (Figure 3D1,D2).

**Figure 4:**
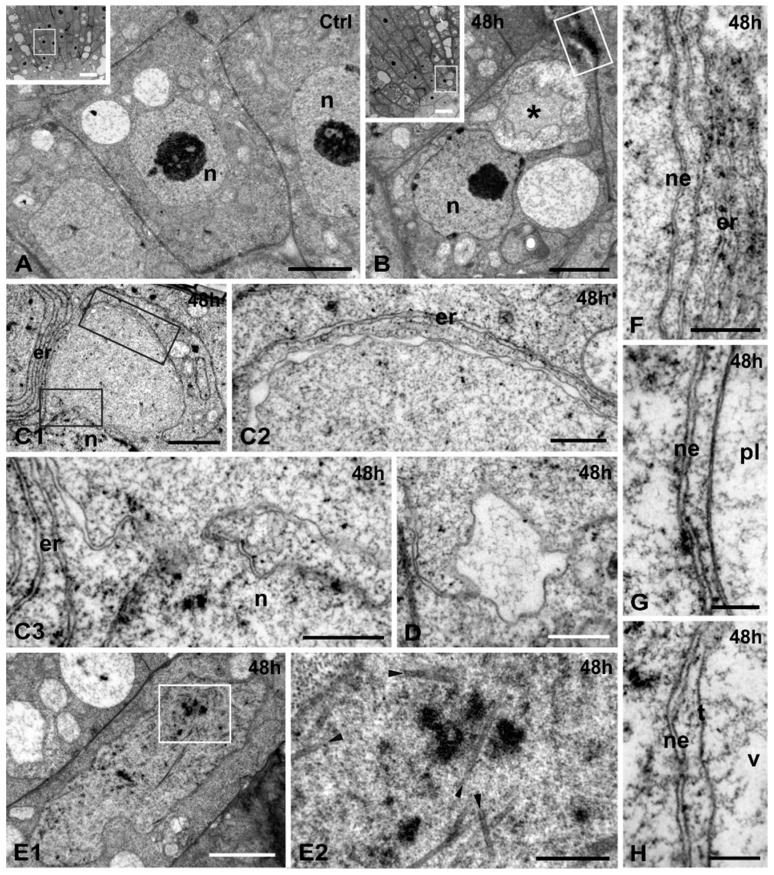
Cr(VI) effects on the nuclear structure and membrane integrity of *A. thaliana*. (**A**,**B**) Cells of untreated (**A**) and 48 h Cr(VI)-treated (**B**) roots, located in the apical meristematic zone (insets, outlined). Nuclei (n) have a smooth contour. In the treated cell (**B**) the nucleus (n) has a wavy contour and one vacuole collapsed (*). Outlined area in (**B**) is magnified in Figure 7E; (**C**) 48 h Cr(VI) treatment. Outlined areas in (**C1**) are enlarged in (**C2**) and (**C3**). A bulbous outgrowth of a nucleus (**C1**), delimited by a double membrane envelope with annulate dilations of the inter-membrane space (**C2**). The nuclear envelope is continuous with the outgrowth membranes in the narrow connecting neck (**C3**); (**D**) A large dilation of an outgrowth inter-membrane space containing fibrous material; (**E**) 48 h Cr(VI) treatment. Outlined area in (**E1**) is magnified in (**E2**); An elongate lobed nucleus (**E1**) bearing several intranucleoplasmic tubular structures of 35–36 nm diameter (**E2**, arrowheads); (**F**–**H**) 48 h Cr(VI) treatment. Proximity of nuclear envelope (ne) with ER (**F**, er), a plastid (**G**, pl) and a vacuole (**H**, v): all membranes are parallel to each other, not contacted and intact. t, tonoplast. Scale bars: Insets: 10 μm; (**A**,**B**,**C1**,**E1**): 2 μm; (**C2**,**C3**,**D**,**E2**): 0.5 μm; (**F**–**H**): 0.2 μm.

The concurrent activity of peroxidase, a H_2_O_2_ scavenger, in similarly treated roots was visualized with pyrogallol staining. In control roots very low peroxidase activity was detected (Figure 3E), but under Cr(VI) stress a time-dependent increased activity of the enzyme was noted (Figure 3F–H). Interestingly, the highest activity of peroxidase was localized in the root tissues of the undifferentiated root zone as confirmed by centrally focused higher magnifications under differential interference contrast optics (Figure 3I–K). These data indicated that peroxidase activity coincided with the higher concentrations of H_2_O_2_ (Figure 3).

### 2.2. Ultrastructural Malformations

TEM revealed a plethora of ultrastructural changes. Low magnification views of central longitudinal sections revealed a generalized vacuolation of Cr(VI)-treated root tip cells (Figure 4A,B, insets). Nuclei of most affected cells did not differ significantly from untreated ones except for their wavy outline (Figure 4A,B). However, some nuclei bore large bulbous outgrowths (Figure 4C1), containing amorphous granular material but not chromatin, and were confined by a double membrane envelope bearing annulate dilations of the inter-membrane space (Figure 4C2). These outgrowths were connected with the main body of the nucleus via a narrow “neck”, where membrane continuity was clearly visible (Figure 4C3). Occasionally, some dilations of the outgrowth membranes were largely distended and contained fibrous material (Figure 4D).

Less frequently, some nuclei appeared larger than usually, elongated and lobed. In the nucleoplasm, several tubular structures were observed among the chromatin (Figure 4E1). Their diameter was 35–36 nm (Figure 4E2).

Several cytoplasmic components such as ER, plastids and vacuoles were located adjacent to the nuclei. ER cisternae, the plastid envelope and tonoplast were lain parallel to the nuclear envelope and intact (Figure 4F–H). The ER cisternae had a similar structure with the nuclear envelope (Figure 4C2,F), while the heterogenous membrane systems were never inter-connected (Figure 4F–H).

**Figure 5:**
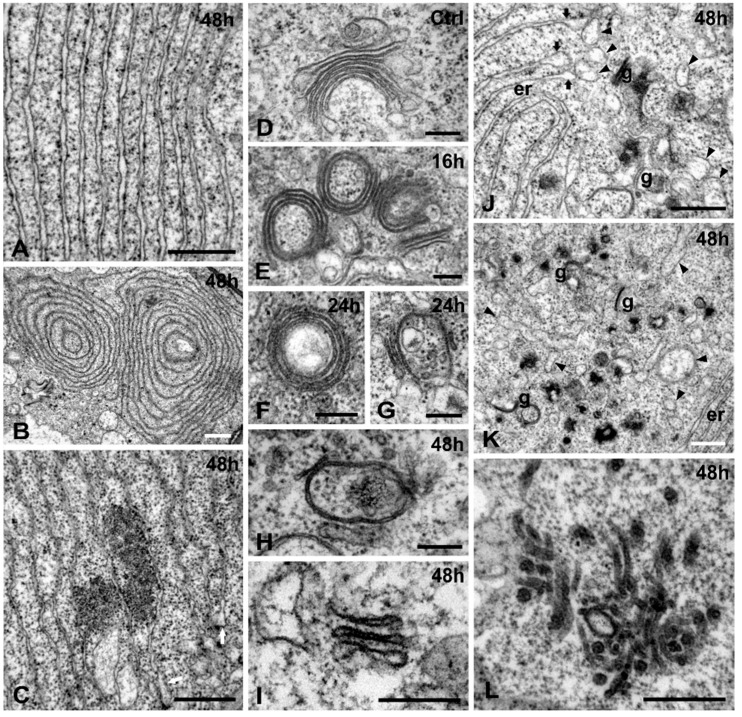
Cr(VI) effects on the endomembrane system of *A. thaliana*. (**A**–**C**) 48 h Cr(VI) treatment, effects on the ER. A bundle of parallel straight ER cisternae (**A**) and concentric conformations of ER (**B**). Amorphous electron dense material in the inter-cisternal spaces of ER and some bulging edges (arrows) (**C**); (**D**–**I**) Cr(VI) effects on the Golgi apparatus; (**D**) Control; (**E**) 16 h Cr(VI) treatment. Golgi bodies with circular or semi-circular conformation. (**F**,**G**) 24 h Cr(VI) treatment. A Golgi body with circular cisternae and translucent enclosure (**F**) and one with reduced cisternae (**G**); (**H**,**I**) 48 h Cr(VI) treatment. Two degenerated Golgi bodies with a cyclic cisterna (**H**) and slightly dilated short cisternae (**I**); (**J**–**L**) 48 h Cr(VI) treatment; (**J**,**K**) Accumulation of Golgi remnants (g) and helical or elongate vesicles (arrowheads). Some vesicles seem to bulge from the edges of ER (er) cisternae (arrows); (**K**) Cytoplasmic area dominated by vesicles, vesicular cisternae, remnants of Golgi bodies and amorphous dense material; (**L**) A clustering of tubular and vesicular structures with dense contents. Scale bars: (**A**,**C**,**J**–**L**): 0.5 μm; (**B**): 1 μM; (**D**–**I**): 0.2 μm.

The endomembrane system was also variously affected by Cr(VI). ER formed large aggregations of parallel, ribosome-bearing, non-swollen cisternae (Figure 5A) or huge concentric conformations (Figure 5B). Sometimes, amorphous electron dense material was encountered in the cytoplasm, between ER cisternae, while frequently the ER cisternae bore bulges (Figure 5C).

Golgi bodies were plentiful in untreated cells, typical in structure, with apparent *cis*→*trans* polarity, surrounded by numerous vesicles (Figure 5D). The Golgi cisternae were frequently curved, yet not closed to rings. In short Cr(VI) exposures (16 h), Golgi bodies displayed normal, circular or semi-circular conformation, enclosing cytoplasmic material (Figure 5E). After 24 h of treatment, many Golgi bodies exhibited a ring-like appearance, enclosing translucent material (Figure 5F), while others were composed of fewer, apparently degenerated cisternae (Figure 5G). In cells treated for 48 h Golgi bodies were severely disorganized, consisting of a single circular cisterna, cisternal remnants (Figure 5H) or stacks of few, loose, short and somewhat swollen cisternae (Figure 5I).

Several cytoplasmic regions of Cr(VI)-treated cells abounded with translucent or dense vesicles, swollen cisternae and remnants of presumable Golgi origin (Figure 5J,K). Some ER cisternae bore swollen ends, giving the impression as if they detach off, generating the nearby population of vesicles (Figure 5C,J). Some helical or elongate vesicles may have derived from larger portions of ER cisternae (Figure 5J,K). Moreover, other areas were dominated by long structures with electron dense contents and smooth outline (Figure 5L).

**Figure 6:**
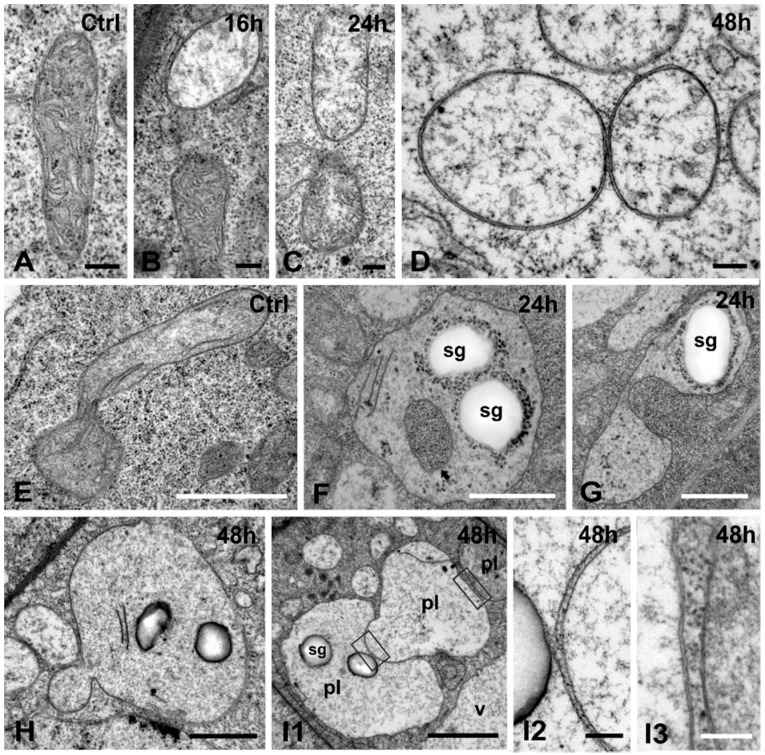
Cr(VI) effects to cytoplasmic organelles of *A. thaliana*. (**A**–**D**) Effects on mitochondria; (**A**) Control. A typical mitochondrion; (**B**) 16 h treatment. One translucent and one normal-looking mitochondrion; (**C**) 24 h treatment. Two mitochondria with translucent and almost translucent appearance; (**D**) 48 h treatment. Mitochondria with transparent stroma and delimited by intact double-membrane envelopes; (**E**–**K**) Cr(VI) effects to plastids; (**E**) Control. A phallus-like plastid bearing a few stroma thylakoids; (**F**,**G**) 24 h treatment; (**F**) An isodiametric plastid containing two starch grains (sg) surrounded by numerous electron dense granules, a few thylakoids (arrowheads) and a cytoplasmic invagination (arrow); (**G**) A saddle-shaped plastid bearing a starch grain (sg) and numerous electron dense granules, centrally engulfed by a deep cytoplasmic invagination; (**H**) 48 h treatment. A plastid having a bulbous expansion in the cytoplasm; (**I**) 48 h treatment. Three plastids (pl), the one intimately protruding toward the neighboring (**I1**). Enlargements of the areas outlined in I1, showing single membranes at the bulging outgrowth (**I2**) and double membrane envelopes of the neighboring plastids (**I3**). v, vacuole. Scale bars: (**A**–**D**,**I2**,**I3**): 0.2 μm; (**E**–**I1**): 1 μm.

Mitochondria of untreated cells were pheiomorphic, delimited by a clearly discernible double membrane envelope and containing numerous cristae in a dense stroma (Figure 6A). In Cr(VI)-treated cells mitochondria progressively exhibited oval to spherical shapes, lost their cristae and exhibited translucent to transparent stroma (Figure 6B–D). In moderate treatments (16, 24 h) affected and non-affected organelles co-existed (Figure 6B,C), illustrating a gradual damage. In longer treatments (48 h), all mitochondria appeared almost transparent and contained granular material of similar structure to the surrounding cytoplasm, confined by intact double membrane envelopes (Figure 6D).

Root tip cells of untreated seedlings bore typical small-sized, pleiomorphic plastids with rudimentary thylakoids and a uniform dense stroma (Figure 6E). Starch grains occurred in root cap plastids, but normally they were absent or very scarce in other root tip cells. In Cr(VI)-exposed cells, however, most plastids had spherical shape and contained unusual inclusions such as starch grains and dense droplets scattered in the stroma or accumulated around the starch grains (Figure 6F). Some plastids bore cytoplasmic invaginations (Figure 6F), which in longitudinal sections exhibited a “saddle” shape (Figure 6G). Other plastids displayed bulging outgrowths within the cytoplasm (Figure 6H) or toward other organelles, including plastids (Figure 6I1). The stroma contained a few internal membranes and was filled with granular material but was less dense than in the control (Figure 6H,I1; *cf.* Figure 6E). At the bulging outgrowths (Figure 6I1), each plastid bore a single membrane (Figure 6I2). Despite the degenerated interior, the plastids were surrounded by intact double membrane envelopes, in which membranes were very close to each other but not fused (Figure 6I3).

**Figure 7:**
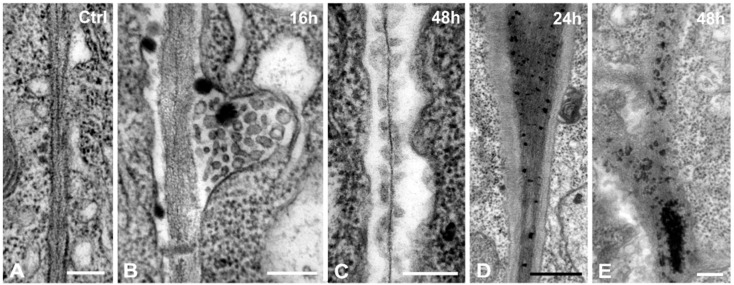
(**A**–**E**) Cr(VI) effects to the cell wall. (**A**) Control; (**B**) 16 h treatment. Accumulation of electron dense droplets and numerous vesicles in the plasma membrane/cell wall interspace; (**C**) Bilateral detachment of the plasma membrane. The middle lamella is flanked by a series of moderate dense structures on either side. (**D**,**E**) 24 h (**D**) and 48 h (**E**) treatment. Accumulation of electron dense granules in the middle lamella; (**E**) is a magnification of the outlined area of Figure 4B. (**A**–**C**,**E**): 0.2 μm; (**D**): 0.5 μm.

Primary cell walls of *A. thaliana* were thin with clearly visible middle lamella, flanked by the plasma membranes of adjoining cells (Figure 7A). In Cr(VI)-treated roots the majority of the cell walls were similar in appearance with the untreated cells. However, in the interspace between the cell wall and the plasma membrane electron dense droplets and vesicles were occasionally observed (Figure 7B). In longer treatments (48 h), moderately dense membranous material as the unaffected cell wall was encountered, resembling strings of beads on either side of the middle lamella (Figure 7C). Other cell walls bore numerous electron dense deposits, mostly located in the middle lamella of the cell wall (Figure 7D,E).

**Figure 8:**
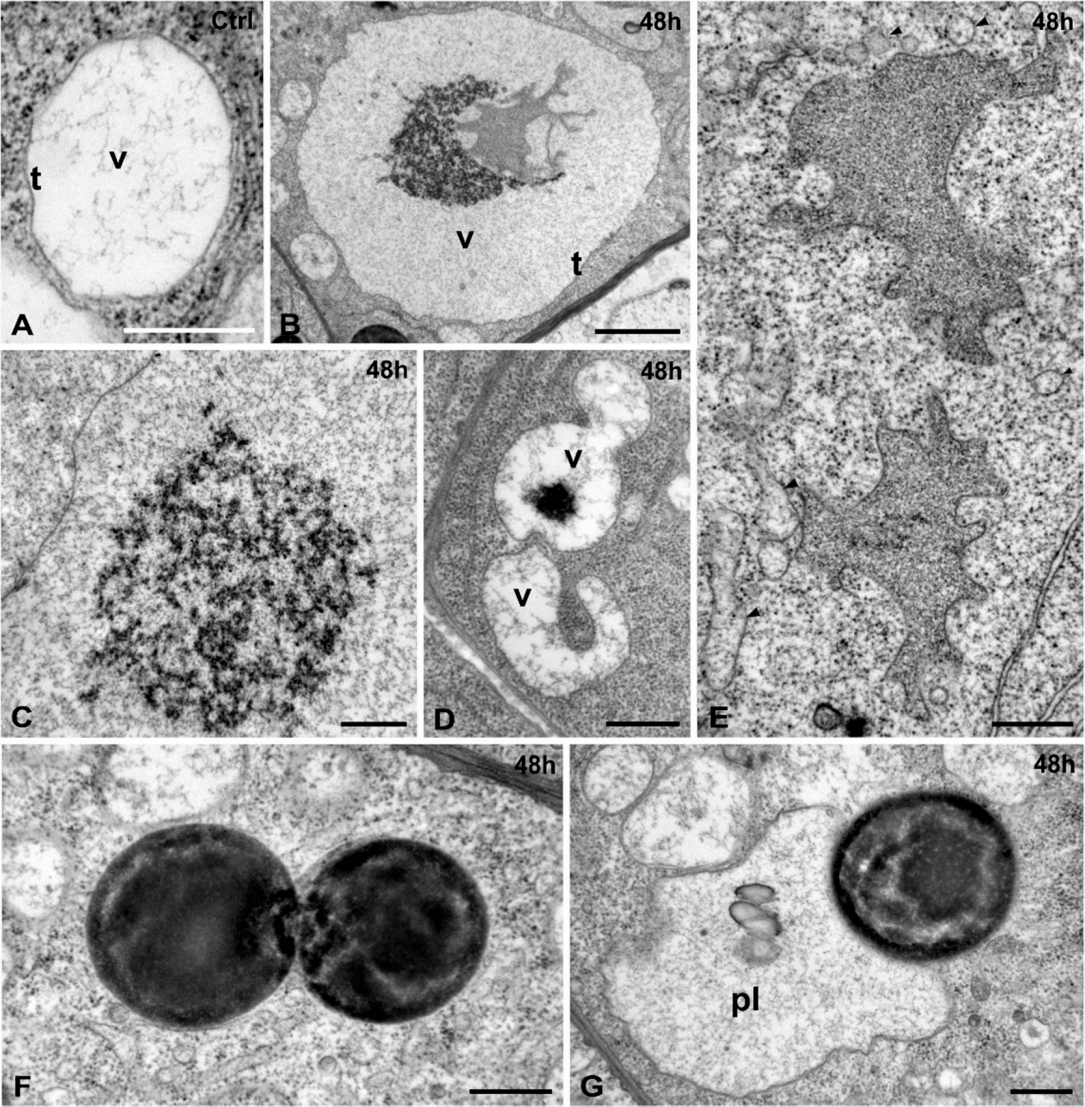
(**A**–**E**) Cr(VI) effects to the vacuoles. (**A**) Control. A small vacuole (v) with smooth tonoplast (t) and a few fibrillar contents; (**B**–**E**) 48 h Cr(VI)-treatment; (**B**) A vacuole with intact tonoplast (t), a central accumulation of electron dense material capping a grayish membrane-bound body with an irregular shape, and fine granular material throughout the vacuole volume; (**C**) Spherical augmentation of granular material within a vacuole; (**D**) Two vacuoles (v) with unusual shape and contents; (**E**) Two membrane-bound irregular structures containing dense granular material, presumably resulted from vacuoles contraction. They are surrounded by cytoplasmic ribosomes and variously shaped vesicles (arrowheads); (**F**,**G**) 48 h treatment. Endocytoplasmic opaque bodies of spherical shape and uneven density, either approaching each other (**F**) or intimately appressed to a plastid (pl) (**G**). Scale bars: (**A**,**D**,**E**–**G**): 0.5 μm; (**B**): 2 μm; (**C**): 1 μm.

Vacuoles in the control cells were few and small, contained some sparse fibrillar or granular material and were delimited by a readily recognizable tonoplast (Figure 8A). In Cr(VI)-treated cells most vacuoles retained their integrity and bore an intact tonoplast (Figure 8B,C), but they contained several inclusions, the most usual of which were variously shaped aggregations of granular precipitates, located centrally or peripherally in the vacuole (Figure 8B,C). Some irregular, membrane-bound structures, containing very dense granular material, were occasionally found within vacuoles (Figure 8B). The remaining vacuolar volume was occupied by dense fine granules (Figure 8B,C). Other vacuoles contained fewer material but larger electron dense deposits or exhibited unusual shapes (Figure 8D). Collapsed vacuoles were also encountered (Figure 4B), having highly irregular shapes (Figure 8E). They were delimited by an continuous membrane and contained very dense granular material, apparently the result of constricting the initial contents in smaller volume. The space formerly occupied by the vacuole was flooded with ribosomes and smooth vesicular or cisternal elements, presumably of ER origin (Figure 8E).

Spherical structures of various size and of high electron density were encountered in the cytoplasm of Cr(VI)-exposed root tip cells, either isolated or accumulated in small or large aggregations (Figure 8F). Some of them were appressed to plastids (Figure 8G). Since they were not delimited by a membrane, it is assumed they represent oil bodies.

## 3. Discussion

### 3.1. Electron Dense Deposits, Cell Wall and Cell Membranes

Plants have evolved a range of potential mechanisms at the cellular level that might be involved in preventing metals to accumulate in toxic concentrations at sensitive internal sites and in sequestering them in some cellular structures to reduce prejudicial effects [26]. Deposition of electron dense material in subcellular compartments, in particular the cell wall, is a common feature of plants exposed to Cr [15,16,18,21] and to many other toxic metals, including aluminum [27], lead [28] and cadmium [29], and is considered one of the first cellular mechanisms against metal toxicity [26]. Electron dense deposits in root tip cells of Cr(VI)-treated *A. thaliana* seedlings have been localized in the cell walls, the cell wall-plasma membrane interspace, the plastids, inside the vacuoles, among the ER cisternae and in the cytoplasm. A distinct attribute of the present study was the localization of electron dense precipitates primarily in the pectic middle lamella rather than in the cellulosic/hemicellulosic component of the cell wall, similarly to *Allium cepa* root tips exposed to lead [28]. The ability of cell walls to accumulate heavy metals stems from their physical and chemical properties. The primary cell walls of *A. thaliana* and in particular middle lamellae are rich in acid pectins [30,31], to which metals bind much stronger than to hemicelluloses and other wall components [28]. This chemical affinity may play an important role in metal tolerance by preventing toxic metal ions from reaching sensitive intracellular sites [27].

The electron translucent appearance of some cell walls presumably was caused by callose deposition, as indicated by aniline blue staining (Figure 2F,G), largely resembling the new cell walls of *Triticum turgidum* experiencing the influence of aluminum [32]. In lead-treated roots of *Allium cepa* cell walls were irregularly thickened or distended, creating large intercellular spaces [28], a feature not observed in our system.

Chromium-induced oxidative stress may cause lipid peroxidation and severe damage to cell membranes [33]. Indeed, a frequently reported effect of Cr stress is membrane injury [15,16,18,21]. In contrast, in *A. thaliana* and under the conditions of our experimental regime almost all types of membranes remained unaffected, being in accordance with Cr-treated growing pollen tubes of *Actinidia deliciosa* (kiwifruit) where cell components were enveloped by intact membranes [17]. The plasma membrane of plant cells, in particular, may be regarded as the first “living” structure that is targeted by metals and constitutes another defense mechanism against metal toxicity [26]. The plasma membrane may function either by resisting to metal entry or through active efflux to the apoplast, where increasing accumulation of the metal favors its binding to the cell wall components and precipitation. Although a plasma membrane repair and protection mechanism may be activated under stress conditions [34], structural integrity of the plasma membrane does not necessarily mean functional efficiency as evidenced by ion leakage [35]. The time-dependent enhancement of Evans blue absorbance by *A. thaliana* Cr(VI)-treated root tips suggests an increase of membrane permeability and mitigation of functional performance [36]. Intracellularly, the tonoplast provides another site where other mechanisms operate transporting actively metals into the vacuole, thus reducing their concentration within the cytoplasm [26]. The high accumulation of electron dense precipitates in the vacuoles is in accordance with this conclusion.

### 3.2. Ultrastructural Effects and Oxidative Stress

Our results showed clear differences in the degree and extent of Cr(VI) injury among the different plant cell compartments. Plastids, mitochondria, Golgi bodies and vacuoles suffered the most severe structural damage; ER, cytoplasm and membranes the least; nuclei and cell walls displayed intermediate impact. This discrepancy might be explained by a differential distribution of Cr within cell compartments [15], by differences in the intracellular concentration of the respective oxidation states considering that part of Cr(VI) may be reduced to Cr(III) [37], or by the differential ROS production of cell organelles [38,39].

Mitochondria were repeatedly shown to suffer under Cr stressful conditions (see Introduction). Literature survey reveals a broad range of mitochondrial responses in various plant species, different tissues and under diverse treatment administrations [15,16,17,18,19,21], which however do not all match those of our system. Mitochondria of Cr(VI)-treated *A. thaliana* displayed a time-dependent depletion of their matrix and disintegration of cristae, presumably caused by the high ROS production in these organelles [36,39]. Their outlining membranes, however, remained intact.

Plastids were also severely affected by Cr(VI) toxicity. Some malformations such as plastid contraction and accumulation of starch grains and plastoglobuli are common features [15,16,18,19,20], but no attachment points or fused areas of the outer plastid membrane with the plasma membrane, as shown in *Spirodela polyrhiza* [16], were observed in *A. thaliana*. Presumably, the increased accumulation of starch grains may be due to impairment of biochemical processes regarding starch metabolism under Cr stress [40]. Moreover, some unique features were noticed in Cr(VI)-treated plastids of *A. thaliana*, such as the bulbous outgrowths and the high incidence of cytoplasmic invaginations, attributed to their pleiomorphic nature and contraction.

The great majority of nuclei in *A. thaliana* appeared ultrastructurally unaffected by Cr(VI), without displaying chromatin condensation [17] and irregular shape, disruption of nuclear envelope or nucleoplasm thinning [21]. Nevertheless, the bulbous outgrowths observed in some nuclei constitute an unusual response not described previously. Presumably, they arise from an internal pressure and local loosening of the nuclear envelope resulting in the outward distention. The contents of the bulbous outgrowths was structurally similar to the nucleoplasm but without chromatin, suggesting that genetic material was not translocated. Noteworthy, the envelope delimiting this distention was an intact double-membrane, continuous with the nuclear envelope but with annulate or large local dilations.

Cytoplasmic components such as Golgi bodies, vesicles, small vacuoles, mitochondria, ER cisternae, paracrystals and tubulin structures have been observed within the nucleus under toxic conditions [13,32,41,42], where they may be entrapped by the reorganizing nuclear envelope at telophase [41]. Tubular structures with a larger diameter (35–36 nm) than typical microtubules (24–25 nm) have been defined as macrotubules and found in cells under adverse experimental administrations [43,44]. The intranucleoplasmic occurrence of macrotubules in Cr-treated cells of *A. thaliana* is another unusual feature reported for the first time, suggesting that tubulin entrapped in the nucleus may be polymerized to atypical conformations.

In Cr-affected root tip cells of *A. thaliana* the number of vacuoles increased compared to the control, in accordance with previous reports [17,21]. Increased vacuolation may provide additional space for the pollutant sequestration. Indeed, electron dense deposits and granulofibrilar material were frequent within vacuoles, some of which, however, collapsed osmotically. The cytoplasm did not change structurally, in accordance with Cr(VI)-treated *Desmidium* cells [20]. Aggregations of dense vesicles and vesicular cisternae at some cytoplasmic areas may represent lipid accumulations (Figure 5L). Similar oil droplets were found in *Oryza sativa* after aluminum exposure [45].

Metals are generally known to cause dysfunction to the electron transport chain and over-reduction, thereby enhancing ROS production [33]. Moreover, intracellular reduction of Cr(VI) to the less toxic Cr(III) [37] enhances ROS generation [7,8]. Inhibition of root growth of *Allium cepa* at high (25–200 μM) doses of Cr(VI) was correlated with the dose-related increase of ROS generation [10]. Similarly, the Cr(VI)-mediated oxidative stress was correlated with ultrastructural changes in root cells of developing rice seedlings [36]. In our study, the Cr-induced production of ROS, as evidenced by the elevated concentration of H_2_O_2_ in the transition zone, may be responsible for the internal morphological damage of mitochondria, plastids and Golgi bodies. Notably, in the same zone more dead cells were visualized by Evans blue staining, while in the meristematic zone, where a higher activity of peroxidases was localized, the H_2_O_2_ concentration was reduced (Figure 3). The hyperactivity of antioxidant enzymes such as peroxidases plays an important role in protecting plants against Cr toxicity *via* scavenging ROS production [10,46]. In conclusion, the spatial coincidence of Cr(VI)-induced oxidative stress with the majority of ultrastructural malformations in *A. thaliana* indicates a correlation of these phenomena.

## 4. Experimental Section

### 4.1. Plant Material and Preliminary Experiments

Seeds of *Arabidopsis thaliana* L. (Heynh) ecotype Columbia (Col-0) were surface sterilized for 5 min with a 30% (*v*/*v*) bleach solution and kept at 4 °C for 48 h. Afterwards, the seeds were germinated and seedlings were grown in Petri dishes on a solid medium containing 1% (*w*/*v*) phytoagar (Duchefa, Haarlem, The Netherlands) and 2% (*w*/*v*) sucrose in a modified Hoagland’s solution (4 mM KNO_3_, 1 mM Ca(NO_3_)_2_, 2 mM KH_2_PO_4_, 0.3 mM MgSO_4_, 0.09 mM Fe-EDTA), plus trace elements (H_3_BO_3_, MnCl_2_, ZnSO_4_, CuSO_4_, MoO_3_), as previously described [47]. The Petri dishes were placed vertically in a growth chamber under a constant 16 h day/8 h night regime at an ambient temperature of 21 ± 1 °C, with light intensity set at 120 μmol·m^−2^·s^−1^. In a preliminary experiment, four-day-old seedlings of similar size were transplanted in Petri dishes containing the same medium supplemented with 10, 25, 50, 100 and 250 μM of Cr(VI), supplied as potassium dichromate (K_2_Cr_2_O_7_, Cr(VI) for brevity), while control seedlings were placed on media without Cr(VI). For each treatment, including the control, 30 seedlings were used. The length of the developing roots was measured every 24 h for 4 consecutive days and the average difference for each concentration was used to draw the growth rate graphs illustrated in Figure 1B. These preliminary experiments were helpful to determine the most suitable concentration and exposure duration, which were 100 μM for 16, 24 and 48 h, that were further adopted for all experiments, although prolonged treatments were also tested.

All chemicals and reagents were purchased from Sigma (Taufkirchen, Germany), Merck (Darmstadt, Germany) and Applichem (Darmstadt, Germany), unless otherwise stated. Digital images were optimized for contrast and color with Adobe Photoshop CS2 and CS6 software with only linear settings.

### 4.2. Detection of Dead Cells with Evans Blue and Trypan Blue

Evans blue is a stain used to selectively detect dead tissues and cells [48]. Ten roots of each of 16, 24 and 48 h treatments in 100 μM Cr(VI) plus controls were incubated in a 0.25% aqueous Evans blue solution for 15 min at room temperature, according to the protocols of Chen *et al.* [49] and Adamakis *et al.* [42]. The roots were then washed twice with double-distilled water and left in distilled water overnight.

In a concurrent experiment other seedlings treated for 24, 48 and 72 h in 100 μM Cr(VI) were stained with 0.5% (*w*/*v*) aqueous solution of Trypan blue for 5 min, another marker of dead cells [50]. Samples were then washed 3–4 times in a PBS solution.

Whole roots of either experiment were observed and photographed under bright field optics of a Zeiss AxioImager.Z2 microscope (Carl Zeiss AG, Munich, Germany) equipped with a digital AxioCam MRc5 camera (Carl Zeiss AG).

### 4.3. Callose Detection with Aniline Blue

For the visualization of callose synthesis induction, Cr(VI)-treated and untreated (controls) roots were incubated in 0.05% (*v*/*v*) aniline blue solution for 30 min in a 0.07 M K_2_HPO_4_ buffer, pH 8.5 [12]. After 3–4 washings in the same buffer, whole mount roots were examined with the Zeiss AxioImager.Z2 microscope.

### 4.4. Detection of Hydrogen Peroxide Production with DCF-DA

Roots of variously treated and untreated seedlings were incubated in the dark with 25 μM 2,7-dichlorofluorescein diacetate (DCF-DA, Sigma) for 30 min [43] for hydrogen peroxide (H_2_O_2_) detection. DCF-DA is a common marker of oxidants and has an increased sensitivity for H_2_O_2_ [51]. After three consecutive washes with double-distilled water, the specimens were examined under a Nikon D-Eclipse C1 confocal laser scanning microscope (CLSM, Nikon Inc., Tokyo, Japan). Special care was taken to retain the laser beam gain equal among the different treatments. Image recording was conducted with the EZ-C1 3.20 software (Nikon Inc.) according to the manufacturer’s instructions.

### 4.5. Detection of Peroxidase Activity with Pyrogallol

To determine the Cr(VI) effects on the peroxidase levels, variously treated root tip cells were immersed in 0.2% (*w*/*v*) pyrogallol (C_6_H_3_(OH)_3_, Sigma-Aldrich, Darmstadt, Germany) solution containing 0.03% (*v*/*v*) H_2_O_2_ in a 10 mM phosphate butter pH 7, for 15 min, at room temperature. After 15 min incubation, oxidation of pyrogallol was detected by the reddish coloration of the tissue [52]. Roots were then washed twice in double-distilled water and whole mounts were studied and photographed with the Zeiss AxioImager.Z2 microscope both under bright field and differential interference contrast optics.

### 4.6. Light and Transmission Electron Microscopy

Roots from Cr-treated and untreated seedlings were conventionally fixed for both light and electron microscopy in 2% (*w*/*v*) paraformaldehyde and 3% (*v*/*v*) glutaraldehyde in 50 mM sodium cacodylate buffer, pH 7.0, supplemented with 5 mM CaCl_2_, as was previously described [44], with modifications. The fixative was poured on the seedlings directly in the Petri dishes, which were gently rocked for 3 h at room temperature. After rinsing in buffer thrice, 15–20 root tips from each treatment of 2–3 mm length were excised, transferred in 5 mL vials and post-fixed in 1% (*w*/*v*) OsO_4_ in the same buffer for 3 h. Following a 3 × 15 min washing in buffer the samples were left overnight in a refrigerator. Then they were dehydrated in a graded acetone series and propylene oxide at 5 °C, embedded in Durcupan ACM (Fluka Chemie AG, Buchs, Switzerland) and sectioned in a Reichert-Jung Ultracut E ultramicrotome (Reichert-Jung Optical Company, Veina, Austria). Semi-thin sections (0.5–1 μm) cut with glass knives were stained with 0.5% (*w*/*v*) toluidine blue O and served for preliminarily observation by light microscopy. Median longitudinal ultrathin sections (80–90 nm) were cut with a diamond knife, collected on copper grids covered with a formvar film, double-stained with uranyl acetate and lead citrate and examined with a JEOL JEM 1011 transmission electron microscope (TEM, JEOL, Ltd., Tokyo, Japan) equipped with a Gatan ES500W digital camera (Gatan Inc., Tokyo, Japan). TEM images were obtained with Digital Migrograph 3.11.2 software (Gatan Inc.).

## 5. Conclusions

Investigation of the effects of Cr(VI) to the fine structure of *A. thaliana* root tip cells revealed a differential injury among the various cell compartments. Plastids, mitochondria, Golgi bodies and vacuoles were the most damaged, ER, cytoplasm and membranes the least, while nuclei and cell walls were intermediately affected. The destruction of the mitochondrial matrix and disintegration of cristae may be interpreted by the high percentage of ROS produced in these organelles [36,39]. The increased accumulation of starch grains within plastids may result from impairment of biochemical processes regarding starch metabolism [40]. Since most of the Cr(VI)-induced ROS production occurs in the cytoplasm, organelles such as Golgi bodies were highly damaged and vacuoles collapsed. Besides, some unique features in *A. thaliana* cells experiencing Cr(VI) stress were observed, including the bulbous outgrowths of nuclei and plastids, the intranucleoplasmic occurrence of macrotubules, vesicular structures budding at the edges of ER cisternae and the formation of lipid droplets in the cytoplasm or in close association with plastids. Sequestration of Cr in the form of electron dense precipitates encountered primarily in the cell wall and secondarily in internal compartments such as vacuoles and plastids, is considered part of a defense mechanism against metal toxicity. On the other hand, all types of membranes appeared structurally intact. Membrane integrity, in particular of plasma membrane and tonoplast, may be regarded as another cellular mechanism to cope with metal toxicity [26], although their functional performance may have been mitigated. It is concluded that the Cr(VI)-induced ultrastructural changes of *A. thaliana* root tips are correlated with increased oxidative stress [36,46] as evidenced by the elevated concentrations of H_2_O_2_, and that the subcellular malformations are organelle specific.
